# DON’T JUST GO THROUGH LIFE, GROW THROUGH LIFE: RESILIENCE AND ITS EFFECT ON SUCCESSFUL AGING

**DOI:** 10.1093/geroni/igz038.2641

**Published:** 2019-11-08

**Authors:** Alexandria G Nuccio, Ashely M Stripling

**Affiliations:** 1 Nova Southeastern University, Davie, Florida, United States; 2 Nova Southeastern University, Fort Lauderdale, United States

## Abstract

Resilience is commonly recognized as a protective factor that increases an individual’s ability to adapt positively to adversity or change. Given the rates of change, which occur in mid and late life, understanding resilience within this population is of particular importance. Yet to date, the direct mechanisms through which resiliency functions remains underexplored in this population. As such, the current investigation seeks to examine the mechanism through which lifelong resiliency may affect the lives of older adults in order to promote successful aging. Data including assessments of lifelong resiliency (measured by an individual’s opinion that life has been a continuous process of learning/changing/growing), environmental mastery (Ryff, 1989), instrumental activities of daily living (IADLs), mood (e.g. depression and self-rated emotional health), and subjective physical health were derived from the Survey of Midlife in the US Database (MIDUS 3). Participants were primarily White/Caucasian (88.7%) and female (54.9%); with a mean age of 63.64 years (SD=11.35). A series of hierarchical multiple linear regressions revealed that resiliency predicted better physical (F=43.448,p<0.001) and emotional health (emotional health (F=46.515,p<0.001), depression (F=17.665,p<0.011)), as well as increased environmental control (IADLs (F=109.992,p<0.001), and Environmental Mastery (F=64.686,p<0.001)) in models adjusted for known correlates (i.e. age, sex, race, marital status, education). The present findings suggest that high levels of resilience increase not only perceptions of the world (subjective environmental mastery, physical and mental health) but also direct outcomes (IADLs). Implications of the current finding include elucidation of the concept of resiliency, as well as, recommendations for increasing resiliency in late life.

